# Improved Antitumor Efficacy and Pharmacokinetics of Bufalin via PEGylated Liposomes

**DOI:** 10.1186/s11671-017-2346-8

**Published:** 2017-11-09

**Authors:** Jiani Yuan, Xuanxuan Zhou, Wei Cao, Linlin Bi, Yifang Zhang, Qian Yang, Siwang Wang

**Affiliations:** 1 Department of Natural Medicine & Institute of Materia Medica, School of Pharmacy, The Fourth Military Medical University, Xi’an, China; 2 Shaanxi Pharmaceutical Development Center, Xi’an, China

**Keywords:** Bufalin, PEGylated liposome, High-pressure homogenization, Pharmacokinetics

## Abstract

Bufalin was reported to show strong pharmacological effects including cardiotonic, antiviral, immune-regulation, and especially antitumor effects. The objective of this study was to determine the characterization, antitumor efficacy, and pharmacokinetics of bufalin-loaded PEGylated liposomes compared with bufalin entity, which were prepared by FDA-approved pharmaceutical excipients. Bufalin-loaded PEGylated liposomes and bufalin-loaded liposomes were prepared reproducibly with homogeneous particle size by the combination of thin film evaporation method and high-pressure homogenization method. Their mean particle sizes were 127.6 and 155.0 nm, mean *zeta* potentials were 2.24 and − 18.5 mV, and entrapment efficiencies were 76.31 and 78.40%, respectively. In vitro release profile revealed that the release of bufalin in bufalin-loaded PEGylated liposomes was slower than that in bufalin-loaded liposomes. The cytotoxicity of blank liposomes has been found within acceptable range, whereas bufalin-loaded PEGylated liposomes showed enhanced cytotoxicity to U251 cells compared with bufalin entity. In vivo pharmacokinetics indicated that bufalin-loaded PEGylated liposomes could extend or eliminate the half-life time of bufalin in plasma in rats. The results suggested that bufalin-loaded PEGylated liposomes improved the solubility and increased the drug concentration in plasma.

**Electronic supplementary material:**

The online version of this article (10.1186/s11671-017-2346-8) contains supplementary material, which is available to authorized users.

## Background

Cancer diseases are of enormous global significance as the population of cancer patients which annually increase may grow by half by 2020 [1]. Due to the existence of the blood brain barrier (BBB) and multidrug resistance, glioma tumor is one of the most life-threatening diseases without effective therapeutic agents clinically [2]. Bufalin has been isolated and identified from *Venenum Bufonis*, which are the secretions of the skin and parotid venom glands of the toad *Bufo bufo* *gargarizans* Cantor or *Bufo melanostictus* Schneider [3]. It has been reported to have strong pharmacological effects including cardiotonic, antiviral, immune-regulation, and especially antitumor effects [4–7]. However, the poor solubility makes it difficult to disperse in the aqueous solution and restricts application [8].

Liposomes have been regarded as a new drug delivery system to improve poor drug solubility in hydrous solution, enhance the bioavailability, increase the therapeutic efficiency, and reduce the side effects [9]. Chiefly, it can be helpful for agents loaded to pass through BBB and delivery to the brain [10]. However, one of the major shortcomings of liposomal formulation is its rapid clearance from the blood due to the absorption of plasma protein to the phospholipid membrane of liposomes, which afterwards triggers the recognition and uptake on the liposomes by the mononuclear phagocytic system. Fortunately, when polyethylene glycol (PEG) is modified on the surface of liposomes, this kind of phagocytose can be sluggish. Hence, it is necessary to study bufalin-loaded PEGylated liposomes as long-circulating liposomes to increase its aqueous solubility and improve its pharmacokinetics [11].

Up to date, studies on the pharmacokinetics of bufalin have not yet been paid much attention. Some reports only focus on the pharmacokinetics of free bufalin in aqueous solution per os administration. In the present study, we developed PEGylated liposomes as a delivery system of bufalin and compared the pharmacokinetic difference among bufalin-loaded PEGylated liposomes, bufalin-loaded liposomes, and bufalin entity in aqueous solution by intravenous administration in rats.

## Methods

### Chemicals and Reagents

Bufalin (≥ 98% in purity) was purchased from BaoJi Chenguang Technology Development Co., Ltd. (Baoji, Shaanxi, China). L-α-phosphatidylcholine, cholesterol, and 1,2-distearoyl-sn-glycero-3-phosphoethanolamine-N-[methoxy(polyethylene glycol)-2000] (ammonium salt; DSPE-PEG_2000_) were purchased from Sigma Chemical Co., Ltd.(St. Louis, MO, USA) (the molecular formulas of these substances were shown in Additional file 1: Figure S1). The acetonitrile used was of spectroscopic grade and purchased from Honeywell (America). Chloroform and alcohol (analytical grade) were purchased from Tianjin Kemiou Chemical Reagent Co., Ltd. (China). All chemicals were analytical or high-performance liquid chromatography (HPLC) grade. And the water was deionized using Millipore water purification system (Milford, MA, USA) and filtered with a 0.22-μm membrane.

### Animals and Cells

This study was carried out in strict accordance with the recommendations in the Guide for the Care and Use of Laboratory Animals of the National Institutes of Health. The protocol was approved by the Institutional Animal Care and Use Committee of Fourth Military Medical University (Shaanxi, China) (approval 2015-1013-R). All surgery was performed under sodium pentobarbital anesthesia, and all efforts were made to minimize suffering. Male Sprague-Dawley rats, initially weighing 250 ± 20 g, were obtained from the Fourth Military Medical University (Xi’an, China). SW620, PC-3, MDA-MB-231, A549, U251, U87, and HepG2 cell lines were respectively purchased from Shanghai Cell Bank or the Experimental Animal Center of the Fourth Military Medical University, cultured in RPMI 1640 supplemented with 10% (*v*/*v*) fetal bovine serum (FBS) and 1% antibiotics (100 U/mL penicillin G and 0.1 mg/mL streptomycin). The cells were maintained in their exponential growth phase in an atmosphere of 5% CO_2_ and 90% relative humidity at 37 °C.

### Synthesis of Bufalin-loaded Liposomes

Liposomes were prepared as a batch size of 10 mL using high-pressure homogenization. The quantity of bufalin used was the same in all liposomes. Briefly, bufalin-loaded common liposomes were prepared with a composition of bufalin, cholesterol, and L-α-phosphatidylcholine in a molar ratio of 10:30:60; bufalin-loaded PEGylated liposomes were synthesized with a composition of bufalin, cholesterol, L-α-phosphatidylcholine, and DSPE-PEG_2000_ in a molar ratio of 10:30:55:5, respectively.

The above phospholipid mixtures were totally dispersed in 3 mL chloroform, and then the chloroform was completely volatilized by a rotary evaporator at 50 °C under decompression condition. Subsequently, residual solvent (if any) was dried by placing in a vacuum desiccator overnight, and then the prepared dry thin film was rehydrated using vortex in 10 mL 50 °C preheated deionized water at 10 mmol/mL phospholipid for 15 min. The mixture thus formed was further processed with high-pressure homogenization. The pressure and time of homogenization was optimized which was found to be 500 bar at 35 °C, and the process has recycled 10 times. The resulting liposome suspension was immediately extruded with a Lipex extruder (ATS, Canada) twice using polycarbonate membranes (0.2-μm pore size, Whatman, Maidstone, UK) to form unilamellar liposomes. Blank liposomal suspension has also been prepared by the same method as described earlier by omitting the step of addition of bufalin.

### Characterization of Bufalin-loaded Liposomes

#### Particle Size and Zeta Potential

The particle size and *zeta* potential of the liposomes were determined by dynamic light scattering technique using the *zeta* potential analyzer (Delsa™ Nano, Beckman Coulter, California, USA). All the bufalin liposomes were diluted with phosphate-buffered saline (PBS) to an appropriate concentration before determining the size distribution and *zeta* potential. The measurements were carried out in the fully automatic mode.

#### High-Resolution Transmission Electron Microscopy

The bufalin liposomes were further characterized with high-resolution transmission electron microscopy (HR-TEM) studies. The bufalin liposomal suspension supported on a 300-mesh copper grid was negatively stained with 1% (*w*/*v*) phosphotungstic acid. The direct imaging of morphology was executed at 200-kV acceleration voltages using a TEM (Hitachi H-7650, Tokyo, Japan).

#### Entrapment Efficiency

The bufalin content was analyzed by HPLC method. The Shimadzu HPLC system (Kyoto, Japan) was equipped with an LC-20AT pump, SPO-M20A diode array detector, CTO-10AS VP column oven, and SIL-10AF autosampler. All separations were carried out on a SinoChrom ODS-BP C18 column (250 mm × 4.6 mm, 5 μm, Yillite) (Yillite, Dalian, China). The injection volume was 20 μL, and the column effluent was monitored at 296 nm. Data was acquired and processed using ClassVP software. The mobile phase consisted of a mixture of acetonitrile: 0.1% potassium dihydrogen phosphate (with phosphoric acid to adjust the pH value of 3.8) with gradient elution (50:50, *v*/*v*). Chromatography was performed at a flow rate of 1.0 mL/min. For determining the entrapment efficiency, 1.0 mL bufalin liposomal suspension was added to the Sephadex G50 column and eluted with PBS. The bluish portion of the effluent was collected and made up to 10.0 mL (the final volume) by adding PBS. The amount (*W*_1_) of bufalin in the liposomal suspension was determined by HPLC method. PBS was added into the other portion of 1.0 mL bufalin liposomal suspension, so as to reach the final volume up to 10.0 mL, and the amount (*W*_2_) of bufalin contained was determined by the same means. The percentage of bufalin entrapped into bufalin liposomes was calculated by the formula:

$$ \mathrm{Entrapment}\  \mathrm{efficiency}={W}_1/{W}_2\times 100\% $$

### In vitro Stability Trial

The entrapment efficiency tested by the Sephadex chromatography method and the particle size determined by *zeta* potential analyzer were applied to assess the stability profile after storage at 4 °C on days 0, 7, 15, 30, and 90. At the five time points, 200 μL of liposome suspension was aspirated for determining the liposomal leakage ratio. Immediately, free bufalin of 200 μL of liposome suspension was separated using Sephadex G50 column chromatography, and the amount of free bufalin was determined by HPLC method. The liposomal leakage ratio was calculated by the formula:

$$ \mathrm{Liposomal}\  \mathrm{leakage}\  \mathrm{ratio}=\left({W}_0-{W}_X\right)/{W}_0\times 100\% $$

$$ {W}_0:\mathrm{entrapment}\  \mathrm{efficiency}\  \mathrm{tested}\ \mathrm{on}\ \mathrm{day}\ 0 $$

$$ {W}_X:\mathrm{entrapment}\  \mathrm{efficiency}\  \mathrm{tested}\ \mathrm{on}\ \mathrm{days}\ X $$

### In vitro Release of Bufalin

The in vitro release behaviors of bufalin from liposomes were conducted using dialysis bag technique at 37 °C as described previously with some modification. PBS (pH = 7.4) containing 10% fetal calf serum was used as a release media. Free drug was removed from the bufalin liposomes by exhaustive dialysis for 4 h against PBS buffer at 4 °C. Briefly, 1.0 mL of bufalin liposomal suspension was pipetted into a dialysis tube (Spectrumlabs, USA; MWCO 30 kDa) with a gentle horizontal shaking (120 rpm/min) at room temperature. The dialysis tube was kept in 50 mL PBS (pH = 7.4) containing 10% fetal calf serum and temperature was maintained at 37 °C. The dialysate was withdrawn from the medium and replaced with an equal volume of fresh PBS at different time durations, viz. hours 0, 0.5, 1, 2, 4, 6, 12, 24, and 48. The concentration of released bufalin was measured by HPLC as described previously.

### Cytotoxicity

In vitro cytotoxicity studies were carried out using MTT (3-(4,5-dimethylthiazol-2-yl)-2,5-diphenyltetrazolium bromide) assay on several kinds of tumor cell lines including SW620, PC-3, MDA-MB-231, A549, U251, U87, and HepG2. Moreover, U87 and U251 cells were selected to measure cytotoxic effects of bufalin-loaded liposomes and bufalin-loaded PEGylated liposomes on glioma tumor cells. U87 and U251 cells were respectively seeded into 96-well microtiter plates at 1.0 × 10^5^ cells/well and allowed to adhere (37 °C, 5% CO_2_) for 24 h, after which the medium was aspirated and replaced with 0.1 mL fresh medium. Bufalin entity, blank liposomes, blank PEGylated liposomes, bufalin-loaded liposomes, and bufalin-loaded PEGylated liposomes were diluted into complete medium and added to cells in a total volume of 0.1 mL to achieve the desired final concentration; for the control, no test solution was added. The blank liposomes and blank PEGylated liposomes were evaluated to test the toxicity of excipients used in the preparation of bufalin liposomes. After 24 h, cell viability was assessed by incubating in a growth medium containing 5 mg/mL MTT for 4 h at 37 °C. After aspirating the culture medium, the formazan crystals formed were solubilized with 200 μL of organic solvent. The absorbance has been measured on a microplate reader at a wavelength of 570 nm.

### In vivo Pharmacokinetic Study of Bufalin Liposomes Using HPLC Method

The pharmacokinetic study was conducted to evaluate the absorption, distribution, metabolism, and excretion of bufalin from common liposomes or long-circulating liposomes and to seek out the differences of the bioavailability of bufalin, using HPLC technique.

In this experiment, young, healthy adult male Sprague-Dawley rats, weighing 250 ± 20 g, were purchased from the Fourth Military Medical University (Xi’an, China). The rats were housed in well-ventilated cages at room temperature (24 ± 2 °C) and 40–60% relative humidity while on a regular 12-h light-dark cycle. The animals were acclimatized for a minimum 3 days prior to the experiment. Animal procedures were performed in accordance with Fourth Military Medical University Animal Ethics Committee Guidelines.

Bufalin-loaded liposomes, bufalin-loaded PEGylated liposomes, and bufalin in aqueous suspension-assisted solubilization by ethanol were prepared as previously described and then intravenously administered at an equivalent dose of 0.5 mg/kg, respectively. Blood samples were collected from the fossa orbitalis wein of rats under light ether anesthesia into micro-fuge tubes containing heparin as an anticoagulant at 2, 5, 15, 30, 45, 60, 90, 120, 240, 360, 600 min post-dosing and then centrifuged at 3500 r/min for 15 min at 4 °C. The separated plasma was stored frozen at −20 °C before assay.

#### Sample Pretreatment

A simple liquid-liquid extraction method was followed for extraction of bufalin and internal standard from rat blood. To 200 μL, an internal standard solution (20 μL of 100 μg/mL cinobufagin working stock) equivalent to 10.0 μg was added and mixed for 10 s on a cyclomixer, followed by extraction with 3.0 mL of ethyl acetate: petroleum ether, 1:1(*v*/*v*), and mixture. The mixture was vortexed for 5 min, followed by centrifugation for 15 min at 4000 r/min on Sigma 3-16k (Frankfurt, Germany). An aliquot of 3.0 mL of organic layer was separated and evaporated to dryness under nitrogen, and then the residue was redissolved in 200 μL acetonitrile. Twenty microliters of supernatant fluid was injected onto analytical column for analysis after 15-min centrifugation at 12,000 r/min.

#### Pharmacokinetic Analysis

The observed maximum blood concentration (*C*_max_) and the half-life time (*T*_1/2_) were obtained by visual inspection of the experimental data. The data was subjected to non-compartmental pharmacokinetics analysis using Drug and Statistics (DAS 2.1.1) edited by the Chinese Pharmacological Society mathematical pharmacological professional committee for drug clinical evaluation. The data were analyzed according to a two-compartment open model.

### Statistical Analysis

All results were expressed as mean ± SD (*n* = 3), and differences between formulations were compared by one-way analysis of variance (ANOVA), using GraphPad Prism 5 software. *P* < 0.05 denotes significance in all cases.

## Results

### Physicochemical Properties

Film rehydration combined with high-pressure homogenization technology was used for the preparation of bufalin-loaded liposomes and bufalin-loaded PEGylated liposomes. Film rehydration is one of the conventional methods to synthesize liposomes with mature and controlled processes to entrap lipophilic drugs. Besides, the number of homogenization cycles plays key roles to achieve uniform and stable liposomal suspension.

**Fig. 1:**
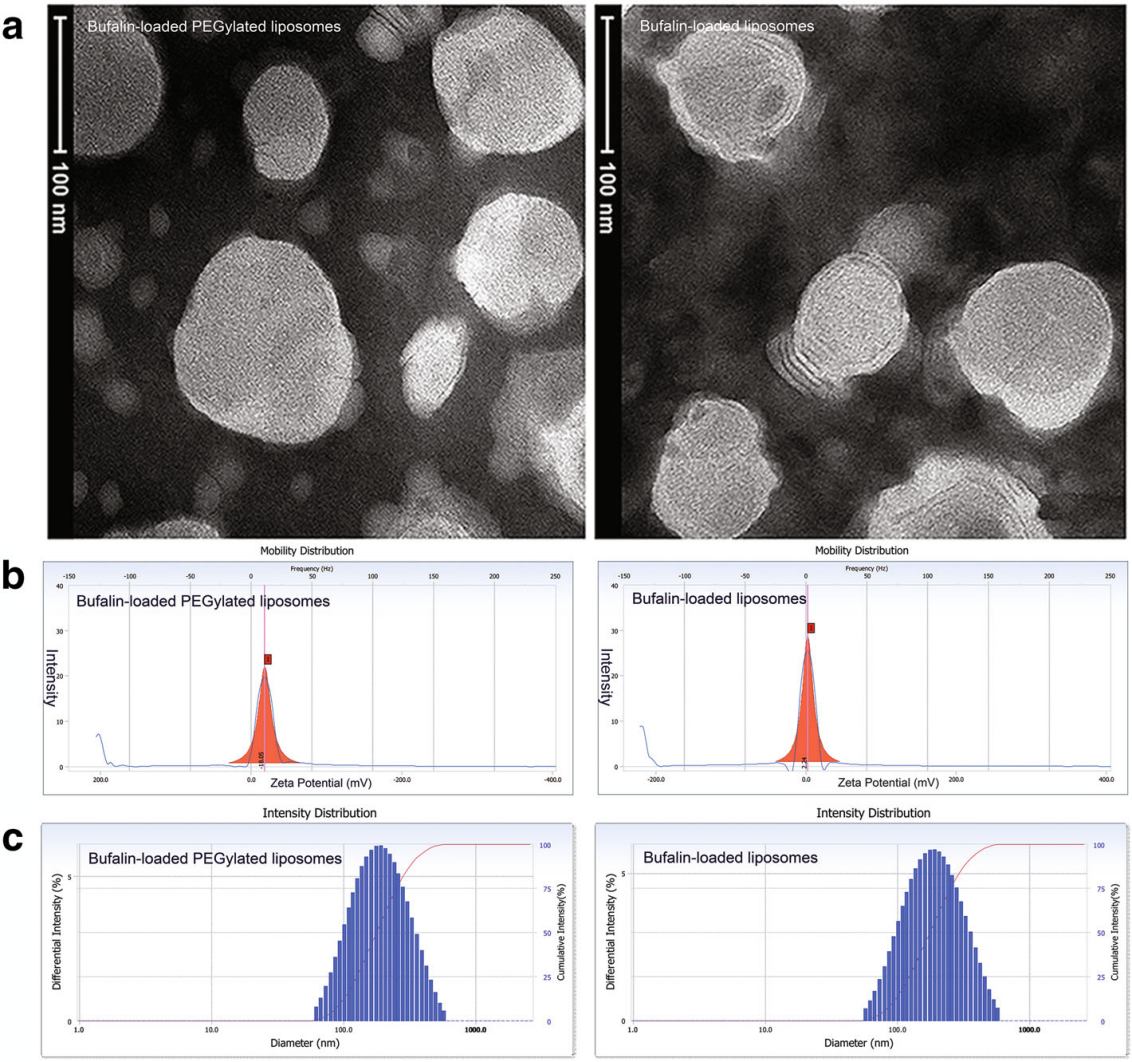
Physicochemical properties of bufalin-loaded liposomes and bufalin-loaded PEGylated liposomes. **a** TEM images of bufalin-loaded liposomes and bufalin-loaded PEGylated liposomes with nearly spherical or oval shape. **b** The typical particle size and distribution of bufalin-loaded liposomes and bufalin-loaded PEGylated liposomes as measured by dynamic light scattering. **c** The typical surface *zeta* potential of bufalin-loaded liposomes and bufalin-loaded PEGylated liposomes

The morphologies of bufalin-loaded liposomes and bufalin-loaded PEGylated liposomes are either nearly spherical or oval as observed by TEM as shown in Fig. 1. According to the TEM image, the mean particle sizes of bufalin-loaded liposomes and bufalin-loaded PEGylated liposomes were both under 200 nm. The mean particle sizes of bufalin-loaded liposomes and bufalin-loaded PEGylated liposomes were 127.6 ± 3.64 nm and 155.0 ± 8.46 nm, respectively (Fig. 1c), measured by *zeta* potential analyzer (Delsa™ Nano, Beckman Coulter, California, USA). Compared with bufalin-loaded liposomes, bufalin-loaded PEGylated liposomes exhibited a negative surface charge, suggesting excellent stability (bufalin-loaded liposomes 2.24 mV; bufalin-loaded PEGylated liposomes −18.05 mV, Additional file 2: Table S1). Moreover, the distributions of *zeta* potential can be seen in Fig. 1b. However, the entrapment efficiency of bufalin in bufalin-loaded liposomes and bufalin-loaded PEGylated liposomes determined by HPLC are 76.31 ± 3.40% and 78.40 ± 1.62%, respectively.

### In vitro Stability Trial

The stability profiles of bufalin-loaded liposomes and bufalin-loaded PEGylated liposomes are shown in Table 1. Moreover, the liposomal leakage ratio also has been calculated. For bufalin-loaded liposomes, the results were respectively 0.0, 16.7, 25.2, 27.9, and 30.6% at 4 °C on days 0, 7, 15, 30, and 90. For bufalin-loaded PEGylated liposomes, the results were respectively 0.0, 5.0, 8.8, 14.5, and 18.0% at 4 °C on days 0, 7, 15, 30, and 90.

**Table 1:** Particle size and entrapment efficiency of bufalin-loaded liposomes and bufalin-loaded PEGylated liposomes in the stability test

Group	Properties	0 day	7 days	15 days	30 days	90 days
Bufalin-loaded liposomes	Particle size (nm)	118.23 ± 5.75	123.47 ± 8.59	125.33 ± 8.45	130.35 ± 3.29	134.58 ± 9.63
	Entrapment efficiency (%)	81.69 ± 1.62	68.08 ± 0.65	61.08 ± 0.65	58.86 ± 1.21	56.70 ± 0.94
Bufalin-loaded PEGylated liposomes	Particle size (nm)	158.30 ± 4.67	153.65 ± 9.46	155.63 ± 8.45	161.83 ± 4.05	168.58 ± 10.47
	Entrapment efficiency (%)	75.33 ± 1.67	71.54 ± 1.14	68.71 ± 1.62	64.43 ± 2.29	61.77 ± 2.73

### In vitro Release Profile

**Fig. 2:**
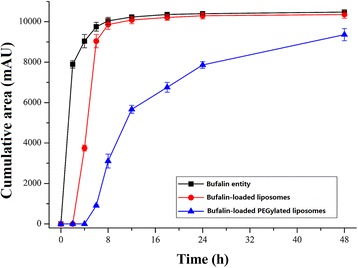
In vitro release of bufalin from bufalin-loaded common liposomes and bufalin-loaded PEGylated liposomes in dissolution media phosphate buffer, pH 7.4. Data are means ± SD, *n* = 3

Bufalin release profile from bufalin-loaded liposomes or bufalin-loaded PEGylated liposomes are summarized in Fig. 2. From the experimental data, in the same dissolution media, bufalin could diffuse into PBS containing 10% fetal calf serum swiftly without restrictions, following with bufalin-loaded liposomes and lastly bufalin-loaded PEGylated liposomes.

### Cytotoxicity

**Fig. 3:**
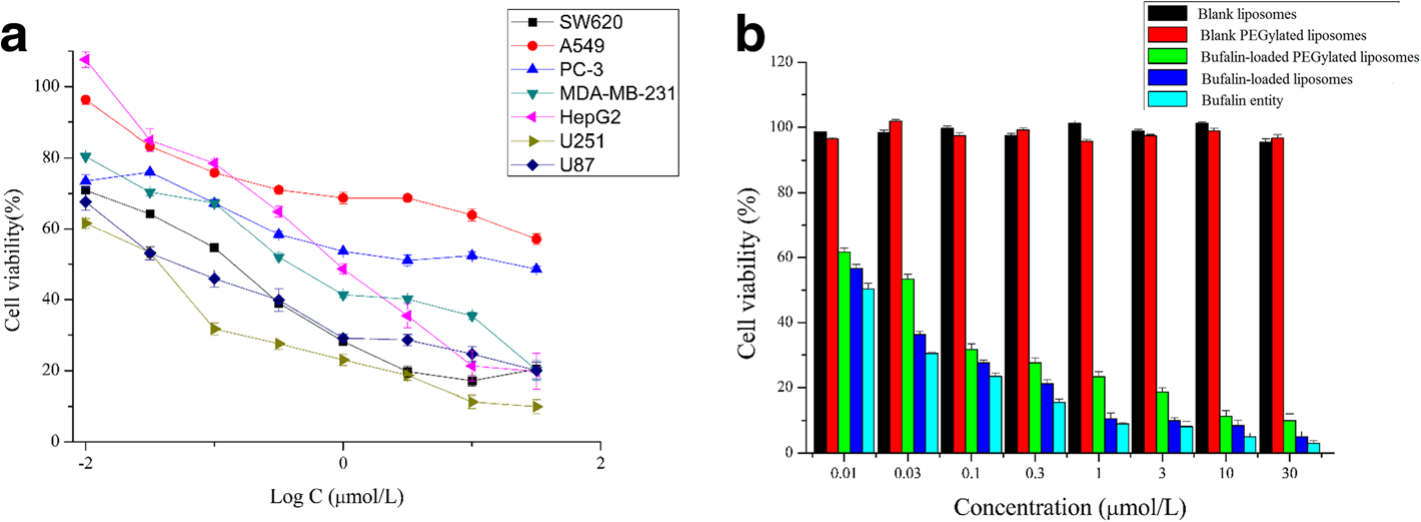
In vitro cytotoxicity of blank liposomes, blank PEGylated liposomes, bufalin entity, bufalin-loaded liposomes, and bufalin-loaded PEGylated liposomes against tumor cells. **a** Cell viability of several kinds of tumor cell lines at various concentrations of bufalin entity. **b** Cell viability of U251 cells at various concentrations of blank liposomes, blank PEGylated liposomes, bufalin entity, bufalin-loaded liposomes, and bufalin-loaded PEGylated liposomes

The cell viabilities of several kinds of tumor cell lines affected by bufalin entity are shown in Fig. 3a when tested by MTT assay, and their half maximal inhibitory concentration (IC_50_) are also calculated in Additional file 2: Table S2. Bufalin caused the inhibition of growth in multiple tumor cells in a dose-dependent manner, while the results of IC_50_ revealed that bufalin was more sensitive to U251 and U87 glioma cancer cells than the other tested cancer cells, respectively. When applied with bufalin entity, bufalin-loaded liposomes, and bufalin-loaded PEGylated liposomes, the cell viabilities of U251 carried out by MTT assay are shown in Fig. 3b. When cultured for 12 h, the cell viabilities of bufalin entity, bufalin-loaded liposomes, and bufalin-loaded PEGylated liposomes exhibited differently, while those of blank liposomes and blank PEGylated liposome did not change. As described, in the same concentration level, cell viabilities of U251 in the bufalin-loaded PEGylated liposome group were always lower than those in the bufalin-loaded liposome group; meanwhile, the impacts of bufalin entity on cell viabilities were minimum.

### In vivo Pharmacokinetic Study

#### Method Validation

The calibration curve was plotted based on linear regression analysis of objection to internal standard peak-area ratio (*y*) versus concentrations (*x*, μg/mL) of bufalin in the standard solution at seven different concentrations. Regression equation was *y* = 0.4238 *x* + 0.2429 (linear range 0.05–10.0 μg/mL), and correlation coefficients (*R* ^2^) were 0.9965. The limit of detection (LOD) value was 0.01 μg/mL, which was calculated as the amount of the injected sample which gave a signal-to-noise ratio of 3 (S/*N* = 3).

#### Specificity, Precision, and Recovery

The degree of interference by endogenous substances was assessed by inspection of chromatograms derived from processed blank plasma samples. Additional file 3: Figure S2 presents typical chromatograms of blank plasma, blank plasma spiked with bufalin, blank plasma spiked with bufalin and resibufogenin (as internal standard), plasma samples spiked with resibufogenin 30 min after intravenous administration of bufalin entity, plasma samples spiked with resibufogenin 30 min after intravenous administration of bufalin-loaded liposomes, and plasma samples spiked with resibufogenin 30 min after intravenous administration of bufalin-loaded PEGylated liposomes. Bufalin and resibufogenin were eluted at approximately 7.191 and 10.131 min, respectively. No peaks of interfering and liposome membrane materials were found at the retention time of bufalin or internal standard. The results of the precision and recovery tests are illustrated in Table 2.

**Table 2:** Results of the precision and recovery test

Concentration (μg/mL)	Intra-day precision			Inter-day precision		
	Mean ± SD (μg/mL)	RSD (%)	Recovery (%)	Mean ± SD (μg/mL)	RSD (%)	Recovery (%)
10.0	10.91 ± 0.36	3.28	109.11	10.09 ± 0.47	4.67	100.85
1.0	1.08 ± 0.05	4.29	108.35	1.00 ± 0.05	4.98	100.22
0.1	0.10 ± 0.003	3.09	99.73	0.098 ± 0.005	4.80	98.06

#### Pharmacokinetics

**Fig. 4:**
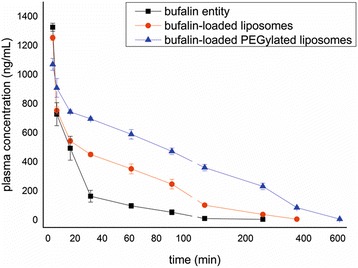
Concentration-time curve of bufalin in plasma in rats

The blood concentration-time profiles of bufalin-loaded liposomes, bufalin-loaded PEGylated liposomes, and its comparison with bufalin entity in aqueous suspension are shown in Fig. 4. The mean pharmacokinetic parameters are presented in Table 3. The results showed that there were significant differences in most of these parameters among them, and the *C*_max_ of bufalin-loaded liposomes was less than the bufalin entity, while that of bufalin-loaded PEGylated liposomes was least. Moreover, the *T*_1/2z_ ratio of bufalin-loaded PEGylated liposomes to bufalin entity and bufalin-loaded liposomes to bufalin entity were about 2.15-fold (87.84 min/40.52 min) (*P* < 0.01) and 1.34-fold (54.40 min/40.52 min) (*P* < 0.05), respectively. The AUC_(0–*t*)_ ratio of bufalin-loaded PEGylated liposomes to bufalin entity and bufalin-loaded liposomes to bufalin entity were about 5.49-fold (139,157.83 ng/(mL min)/25,334.27 ng/(mL min)) (*P* < 0.01) and 2.28-fold (57,751.88 ng/(mL min)/25,334.27 ng/(mL min)) (*P* < 0.01), respectively. It revealed that bufalin entity was rapidly cleared in the bloodstream and liposome preparation increased the drug concentration in plasma and withstood the clearance. Furthermore, modification of PEG could facilitate this effect.

**Table 3:** The main plasma pharmacokinetic parameters of bufalin after intravenous administration of bufalin entity, bufalin-loaded liposomes, and bufalin-loaded PEGylated liposomes in rats (*n* = 6)

Parameters (unit)	Bufalin entity	Bufalin-loaded liposomes	Bufalin-loaded PEGylated liposomes
AUC_(0–*t*)_ (ng/(mL min))	25,012.0 ± 2246.3	57,137.5 ± 1453.1**	138,046.0 ± 2771.8**
AUC_(0–∞)_ (ng/(mL min))	25,334.3 ± 2243.9	57,751.9 ± 1554.0^**^	139,157.8 ± 2984.1^**^
MRT_(0–*t*)_ (min)	29.74 ± 1.39	69.3 ± 2.0^**^	138.1 ± 3.3^**^
MRT_(0–∞)_ (min)	33.3 ± 2.3	73.28 ± 2.8^**^	142.9 ± 3.7^**^
*T*_1/2z_ (min)	40.5 ± 10.6	54.4 ± 3.3^*^	87.8 ± 7.8^**^
*V*_z/F_ (L/kg)	0.003 ± 0.001	0.001 ± 0.0008^**^	0.0002 ± 0.0004^**^
*C*_max_ (ng/mL)	1326.6 ± 24.5	1254.0 ± 16.6^**^	1071.3 ± 36.3^**^

*AUC*_*(0–t)*_ area under the curve from 0 to *t*, *AUC*_*(0–∞)*_ area under the curve from 0 to ∞, *MRT*_*(0–t)*_ mean retention time from 0 to *t*, *MRT*_*(0–∞)*_ mean retention time from 0 to ∞; *T*_*1/2z*_ half-life, *V*_*z/F*_ volume of distribution, *C*_*max*_ peak concentration

*P < 0.05, **P < 0.01 compared with bufalin entity group

## Discussion

This study prepared bufalin-loaded liposomes and bufalin-loaded PEGylated liposomes successfully by homogenization-film rehydration method, both of which had optimum size range and low polydispersity and could be easily reproduced in a large batch size. This study found that liposomal formulation improved the solubility of bufalin greatly.

Bufalin-loaded PEGylated liposomes exhibited a negative surface charge with a larger absolute value than the bufalin-loaded liposomes with a positive surface charge. The *zeta* potential is mainly determined by the surface properties. The unmodified bufalin-loaded liposomes were neutral, and their potentials were generally within ± 5 mV. However, DSPE is not charged under neutral conditions, but the preparation process cannot be completely neutral, so DSPE is negatively charged. Meanwhile, the *zeta* potential of macromolecule PEG is about negative for several millivolts, near neutral negative potential. Therefore, after the surface modification of DSPE-PEG_2000_ on bufalin-loaded liposomes, the *zeta* potential of bufalin-loaded PEGylated liposomes is negative. These findings suggest that the surface modification of DSPE-PEG_2000_ changes the surface electric potentials of liposomes, which enhanced their stability. It had been confirmed by in vitro stability test. Li et al. [12] employed a liposome co-delivery system that coupled anti-CD40 mAbs to the surface of PEGylated liposomes wrapped with bufalin. The liposomes were synthesized with a composition of bufalin, cholesterol, EPC, DSPE-PEG_2000_, and DSPE-PEG_2000_-Mal in a molar ratio of 20:55:5:5:15, respectively. Then anti-CD40 monoclonal antibody was conjugated to the liposomes through the maleimide-thiol reaction for the preparation of bufalin liposome-anchored anti-CD40. With regard to the stability profile, it is only mentioned that the negative surface charge suggests excellent stability without clear and definite data description.

Li et al. [13] prepared bufadienolide-loaded liposome (BU-lipo) consisting of Lipoid E-80® 1.25% and cholesterol 0.06%. The entrapment efficiencies of bufalin, cinobufagin, and resibufogenin were 86.5, 90.0, and 92.1%, respectively. Considering the stability of phospholipids, pH 6.5 was chosen for the BU-lipo formulation. The properties of BU-lipo, such as pH, PSD, *zeta* potential, and entrapment efficiency, did not change for at least 3 months at 2–8 °C. However, the storage condition needed to be strictly controlled. A more stable preparation at neutral pH needs to be studied so as to reduce the transport and storage costs, which should be taken into consideration for the future clinical application.

According to the preparation formulation of the previous studies, we replicated the experiments about bufalin-loaded liposomes. However, the stability of liposomes could not meet the production needs.

In the present study, bufalin-loaded PEGylated liposomes were synthesized with a composition of bufalin, cholesterol, L-α-phosphatidylcholine, and DSPE-PEG_2000_ in a molar ratio of 10:30:55:5, respectively. Especially before being extruded with a Lipex extruder using polycarbonate membranes, the mixture thus formed was further processed with high-pressure homogenization.

When stored in neutral pH at 4 °C for 3 months, the particle sizes of both liposomes increased slightly and the entrapment efficiency declined modestly overall, which will be convenient to be used in clinical application. A TEM photograph showed that a thick three-dimensional cloud-like structure covered the surface of bufalin-loaded liposomes, which indicates that DSPE-PEG_2000_ plays a role in steric stabilization owing to its amphiphilic linear polymer structure feature.

An in vitro release study revealed that the releases of bufalin from bufalin-loaded liposomes and bufalin-loaded PEGylated liposomes were delayed in the same dissolution media, while bufalin entity diffused into PBS swiftly without restrictions. The surface modification of DSPE-PEG_2000_ altered the barrier properties of the aqueous boundary layer and the permeability of the membrane, resulting in a low release velocity of bufalin from liposomes.

Regarding cytotoxicity study, bufalin caused an obvious inhibition of growth in multiple tumor cells in a dose-dependent manner, while the results of IC_50_ indicated that bufalin was more sensitive to U251 and U87 glioma cancer cells than the other kinds of cancer cells, respectively. Moreover, we found that the blank liposome and blank PEGylated liposome were not toxic to cells, revealing the safety of excipients to some degree, whereas the bufalin-loaded liposomes and bufalin-loaded PEGylated liposomes showed concentration-dependent toxicity to U251 glioma cells. In addition, bufalin-loaded PEGylated liposomes showed slightly weaker cytotoxicities compared with bufalin-loaded liposomes and free bufalin. It is assumed that free bufalin was slowly released from bufalin-loaded PEGylated liposomes during incubation. And the release rate of bufalin was slower in bufalin-loaded PEGylated liposomes than bufalin-loaded liposomes. All the results might suggest that after internalization in the cells, free bufalin could be released from bufalin-loaded PEGylated liposomes and induce the apoptosis of U251 glioma cells.

The pharmacokinetic study found that significant differences of pharmacokinetic parameters among bufalin-loaded PEGylated liposomes, bufalin-loaded liposomes, and free bufalin entity did exist. After intravenous administration, free bufalin entity was swiftly cleared in the bloodstream with the highest peak concentration, whereas liposome preparation did obviously increase the drug concentration in plasma and withstood the clearance. Moreover, the surface modification of PEG could facilitate this effect. These results had provided us some information for understanding the properties of bufalin on pharmacokinetics and pharmacodynamics.

As common primary intracranial tumors, 70% of glioma tumors were malignant, including astrocytoma and glioblastoma. The micro environment of the network of glioma was composed of tumor cells, immune cells and various cytokines secreted by them, which was complex and made glioma cells intracranial metastatic easily [2]. Therefore, operations are difficult to perform, and the incomplete surgery excision commonly resulted in the poor prognosis. To these patients, radiotherapy and chemotherapy play key roles in their treatments. Nowadays, the applications of three-dimensional conformal radiation therapy and intensity-modulated radiation therapy technology can not only increase precision, dosage and efficacy, but also reduce damage in cancer therapy, so as to improve their living quality. However, the accurate radiotherapy has great risk because of the particularity of brain tissues and brain functions. Hence, medication of chemotherapeutics is relatively instrumental in glioma cancer treatment, whereas most malignant and highly aggressive glioma tumors have high relapse rates due to BBB and drug resistance [14].

Consequently, selecting a highly sensitive drug to brain glioma and making it penetrate the BBB are becoming emerging scientific subjects to resolve.

In recent years, the researches concerning bufalin have been studied in various aspects, including pharmacology, pharmacodynamics, and pharmaceutics [12, 15–20]. Fortunately, some studies found that bufalin had a broad antitumor spectrum. It was sensitive to several kinds of tumor cell lines, including lung cancer, liver cancer, melanoma, and glioma cancer. However, low solubility, heavy toxicity, and short half-life led to a narrow therapeutic index by intravenous administration and easy decomposition by oral administration, which restricted clinical application of bufalin [6]. In addition, bufalin had been reported to be hydrophobic and could only dissolve in some suspending agent or auxiliary solvent including ethanol and tween 80, which might result in some toxic effects [21].

Nevertheless, as a new drug delivery system, the liposomal formulation was considered to be a low-toxic technology with considerable potential for encapsulating lipophilic drugs [12, 22]. Some studies showed that liposome could be easier to enter tumor focus by the process of phagocytosis. Meanwhile, the vascular endothelial cell gaps in the tumor lesion was enlarged, which enhanced this process (enhanced permeability and retention effect, EPR effect) [23, 24]. Moreover, the liposome could sustain releasing antitumor agents so as to extend action time, improve drug efficacy, and decrease adverse reaction [25]. Thus, liposome formulation has a wide prospect to encapsulate antitumor agents [9].

In the previous experiments, we found that the combination chemoimmunotherapy of anti-CD40 plus bufalin by liposomal carriers could enhance anticancer therapeutic efficacy while reducing systemic toxicity, due to the confined biodistribution and prolonged release of cargo [12]. In the present study, we found that bufalin entity has a broad spectrum of anticancer activity. It could inhibit the proliferation of several kinds of tumor cells, such as lung, liver, breast, and glioma cancer cells. As an active traditional Chinese monomer, bufalin is a promising anticancer drug. It was expected to be applied to further clinical study. However, anti-CD40 mAbs has not been approved as a pharmaceutical excipient by the FDA. The toxicity and side effect of anti-CD40 mAbs is still unclear to this day.

Nevertheless, the shortcomings of conventional liposome were obvious in the meantime. Lack of membrane stability, easy oxidation of phospholipid materials, and opsonification of plasma proteins limited its application [26, 27]. By surface-modifying DSPE-PEG_2000_ on bufalin-loaded liposomes, bufalin-loaded PEGylated liposomes extended its blood circulation, prolonged half-life, and expanded the therapeutic window for glioma.

Therefore, we adjusted the formulation of bufalin-loaded PEGylated liposomes. On this basis, we evaluated the physicochemical properties and comprehensive characterizations both in vitro and in vivo. We characterized pharmacokinetic properties of bufalin-loaded PEGylated liposomes and compared this type of liposome with raw bufalin and non-PEGylated liposomes. Our results revealed the unknown PK properties of this type of liposomes, which will be useful to recommend dosage in a further clinical study.

Our subjects lack some tissue distribution experiments in animals, so it is not known how the liposome formulation altered the distribution of bufalin in the body. Further studies with more experiments are needed.

## Conclusions

The present study demonstrated that the solubility, antitumor efficacy, and pharmacokinetics of bufalin-loaded PEGylated liposomes are improved compared with those of bufalin entity. The results suggested that PEGylated liposomal bufalin has the potential to be a good drug delivery system for glioma cancer.

## Additional files

Additional file 1: Figure S1. The molecular formulas of the substances used in the formulation. (JPEG 103 kb)

Additional file 2: Table S1. Characterization of bufalin-loaded liposomes and bufalin-loaded PEGylated liposomes. Table S2. Inhibition of bufalin on six kinds of tumor cell lines. (DOCX 14 kb)

Additional file 3: Figure S2. Characteristic chromatogram of bufalin in plasma. (a) Blank plasma. (b) Blank plasma spiked with bufalin. (c) Blank plasma spiked with bufalin and resibufogenin (as internal standard). (d) Plasma samples spiked with resibufogenin 30 min after intravenous administration of bufalin entity. (e) Plasma samples spiked with resibufogenin 30 min after intravenous administration of bufalin-loaded liposomes. (f) Plasma samples spiked with resibufogenin 30 min after intravenous administration of bufalin-loaded PEGylated liposomes. (1) Bufalin; (2) Resibufogenin. (TIFF 1372 kb)
