# Supplementary figures and images for: Improved Antitumor Efficacy and Pharmacokinetics of Bufalin via PEGylated Liposomes
Source: Nanoscale Res Lett. 2017 Nov 9;12:585. doi: 10.1186/s11671-017-2346-8 (PMC5680394; doi:10.1186/s11671-017-2346-8)

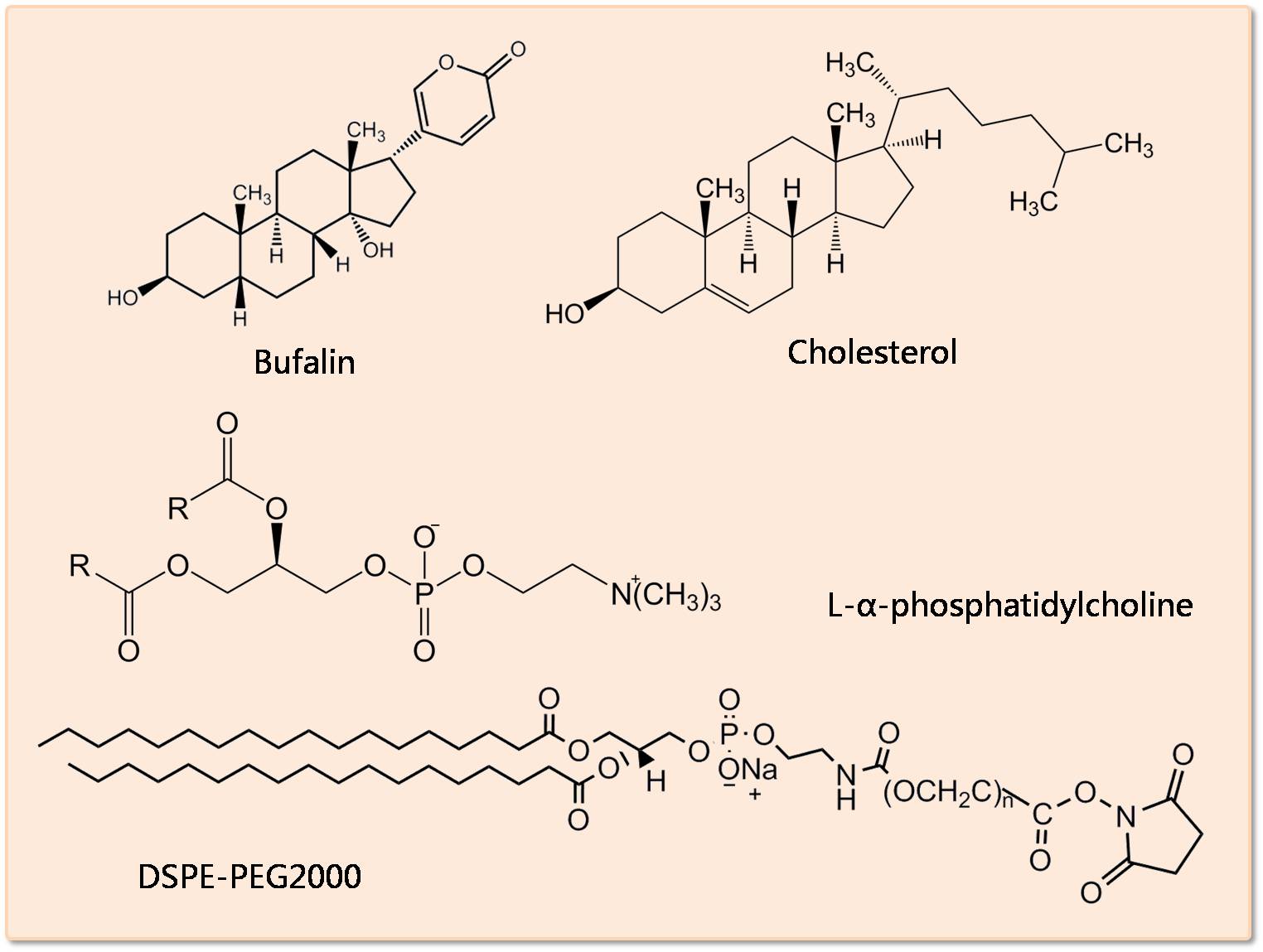

Supplement: Supplementary file 1 — The molecular formulas of the substances used in the formulation. (JPEG 103 kb) [file 11671_2017_2346_MOESM1_ESM.jpg]

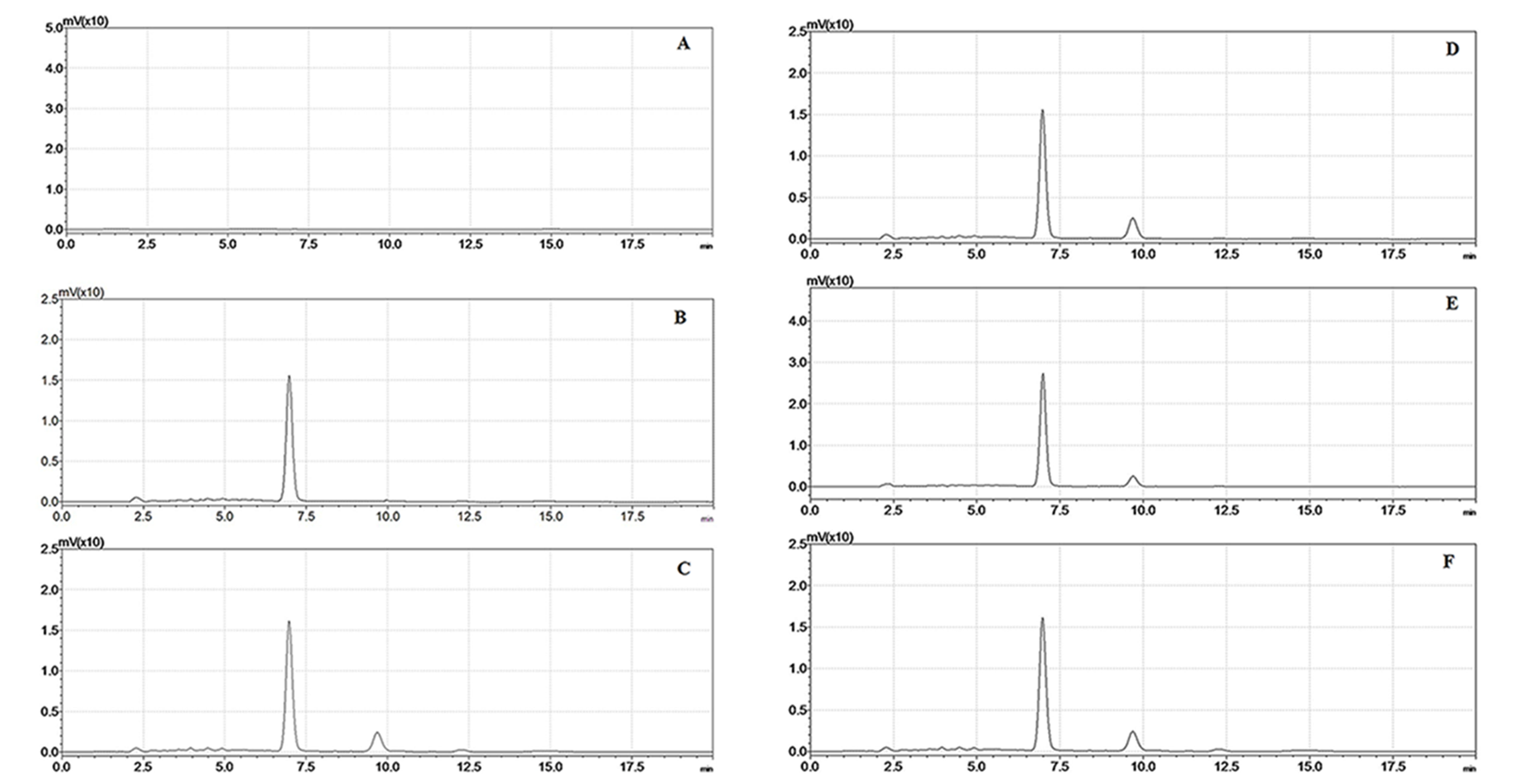

Supplement: Supplementary file 3 — Characteristic chromatogram of bufalin in plasma. (a) Blank plasma. (b) Blank plasma spiked with bufalin. (c) Blank plasma spiked with bufalin and resibufogenin (as internal standard). (d) Plasma samples spiked with resibufogenin 30 min after intravenous administration of bufalin entity. (e) Plasma samples spiked with resibufogenin 30 min after intravenous administration of bufalin-loaded liposomes. (f) Plasma samples spiked with resibufogenin 30 min after intravenous administration of bufalin-loaded PEGylated liposomes. (1) Bufalin; (2) Resibufogenin. (TIFF 1372 kb) [file 11671_2017_2346_MOESM3_ESM.tif]
