# Supplementary material for: Improved Antitumor Efficacy and Pharmacokinetics of Bufalin via PEGylated Liposomes
Source: Nanoscale Res Lett. 2017 Nov 9;12:585. doi: 10.1186/s11671-017-2346-8 (PMC5680394; doi:10.1186/s11671-017-2346-8)
Supplement: Supplementary file 2 — Characterization of bufalin-loaded liposomes and bufalin-loaded PEGylated liposomes. Table S2. Inhibition of bufalin on six kinds of tumor cell lines. (DOCX 14 kb) [file 11671_2017_2346_MOESM2_ESM.docx]

**Table S1** Characterization of bufalin-loaded liposomes and bufalin-loaded PEGylated liposomes

| Liposome formulation | Particle size (nm) | PDI | *Zeta* potential (mV) | Entrapment efficiency  (%) |
| --- | --- | --- | --- | --- |
| bufalin-loaded liposomes | 127.6 ± 3.64 | 0.237 | 2.24 ± 0.57 | 76.31 ± 3.40 |
| bufalin-loaded PEGylated liposomes | 155.0 ± 8.46 | 0.189 | -18.05 ± 4.49 | 78.40 ± 1.62 |

**Table S2. Inhibition of bufalin on 6 kinds of tumor cell lines**

| Cell lines | IC_50_ (μmol/L) |
| --- | --- |
| SW620 | 0.065±0.011 |
| PC-3 | 17.70±1.06 |
| MDA-MB-231 | 0.27±0.02 |
| A549 | 422.82±9.23 |
| U87 | 0.089±0.009 |
| HepG2 | 0.61±0.06 |
| U251 | 0.091±0.005 |
